# Grill Bristle Induced Perforation of the Bile and Pancreatic Ducts Managed by Endoscopic Ultrasound and Endoscopic Retrograde Cholangiopancreatography: A Unique Foreign Body Case

**DOI:** 10.14309/crj.0000000000001873

**Published:** 2025-10-30

**Authors:** Larissa Mercadante de Assis, Abdulrahman Qatomah, Daryl Ramai, Marvin Ryou

**Affiliations:** 1 Division of Endoscopy, Hospital das Clínicas de São Paulo, São Paulo, Brazil; 2 Division of Gastroenterology, Hepatology, and Endoscopy, Brigham and Women's Hospital, Harvard Medical School, Boston, MA; 3 Division of Gastroenterology and Hepatology, King Faisal Specialist Hospital and Research Center, Jeddah, Saudi Arabia; 4 Division of Gastroenterology, Hepatology, and Nutrition, University of Utah, Salt Lake City, UT

**Keywords:** foreign body, common bile duct, pancreas, ERCP, grill brush bristle

## Abstract

Foreign body ingestion is common, but penetration into the common bile duct (CBD) and pancreas is exceptionally rare. A 74-year-old woman presented with 4 weeks of intermittent left upper abdominal pain and normal laboratory results. Computed tomography showed a linear hypodensity traversing the ampulla and pancreatic duct. Endoscopic ultrasound confirmed a hyperechoic linear object in the CBD. Endoscopic retrograde cholangiopancreatography with sphincterotomy and balloon sweeps revealed a metallic foreign body protruding from the papilla. It was removed using pediatric cold forceps and identified as a grill brush bristle. The patient was discharged without complications and remained asymptomatic at 4 weeks. Grill brush bristle ingestion can rarely result in CBD and pancreatic duct penetration. Endoscopic ultrasound facilitates localization, and endoscopic retrograde cholangiopancreatography provides a safe, minimally invasive treatment.

## INTRODUCTION

Foreign body (FB) ingestion is a common occurrence in clinical practice, and in most cases, the object passes through the gastrointestinal tract without complications.^1^ However, diagnosis and management can be challenging and depend on the FB's location, composition, and clinical presentation. We report a rare case of a penetrating FB lodged in the common bile duct (CBD) and extending into the head of the pancreas in a patient with no prior history of papillary manipulation or surgery.

## CASE REPORT

A 74-year-old woman presented to an urgent care clinic complaining of 4 weeks of sharp, constant, and nonradiating intermittent left upper abdominal pain. The pain was not associated with food consumption. Laboratory testing showed normal white cell count, liver function tests, and pancreatic enzymes (including lipase and amylase).

**Figure 1.**
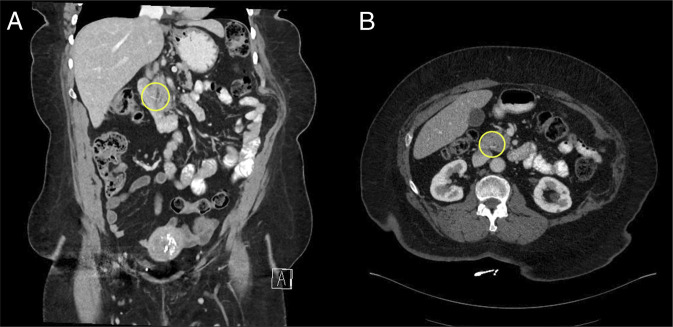
Computed tomography image showing linear hypodensity traversing the wall of the second portion of the duodenum and extending to the pancreatic head and traversing the pancreatic duct (A) coronal plane (B) axial plane.

**Figure 2.**
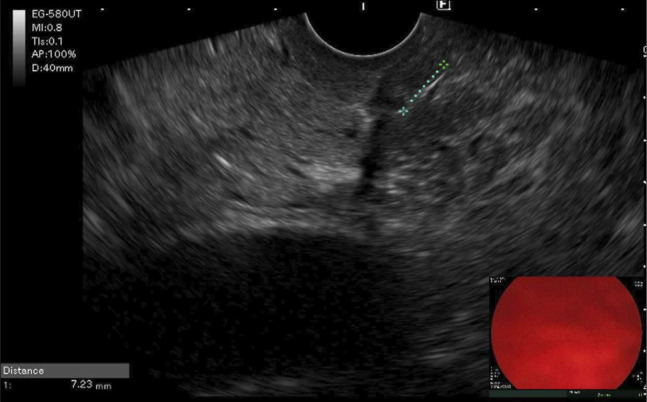
Endoscopic ultrasound image showing a sharp hyperechoic linear object traversing the bile duct.

A computed tomography scan performed 1 week after presentation revealed a linear hypodensity traversing the ampulla and pancreatic duct, extending toward the pancreatic head, suggestive of a lodged FB (Figure 1). The patient was subsequently referred for outpatient upper endoscopy with endoscopic ultrasound (EUS) evaluation (Figure 2).

### Endoscopy

The examination revealed normal papilla and no signs of significant abnormality in the pancreas; however, a hyperechoic linear object was noted traversing the bile duct on endosonographic examination. The decision was made to proceed with endoscopic retrograde cholangiopancreatography (ERCP) for FB removal.

**Figure 3.**
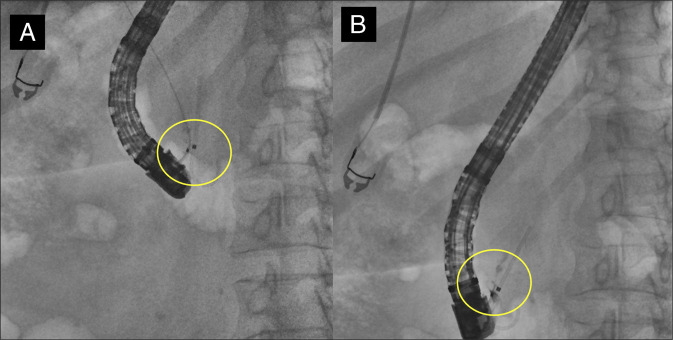
Initial scout film (A) questionable linear object in the right upper quadrant (B) possible filling defect in the lower third of the biliary tree.

**Figure 4.**
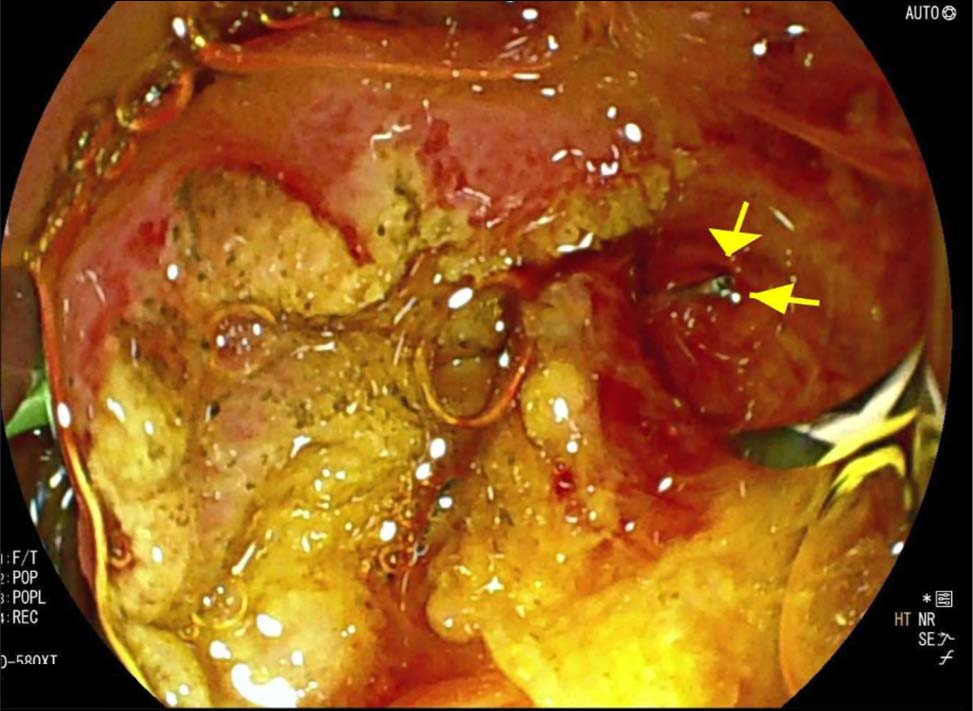
Endoscopic retrograde cholangiopancreatography image showing a sharp foreign body emerging from the bile duct.

**Figure 5.**
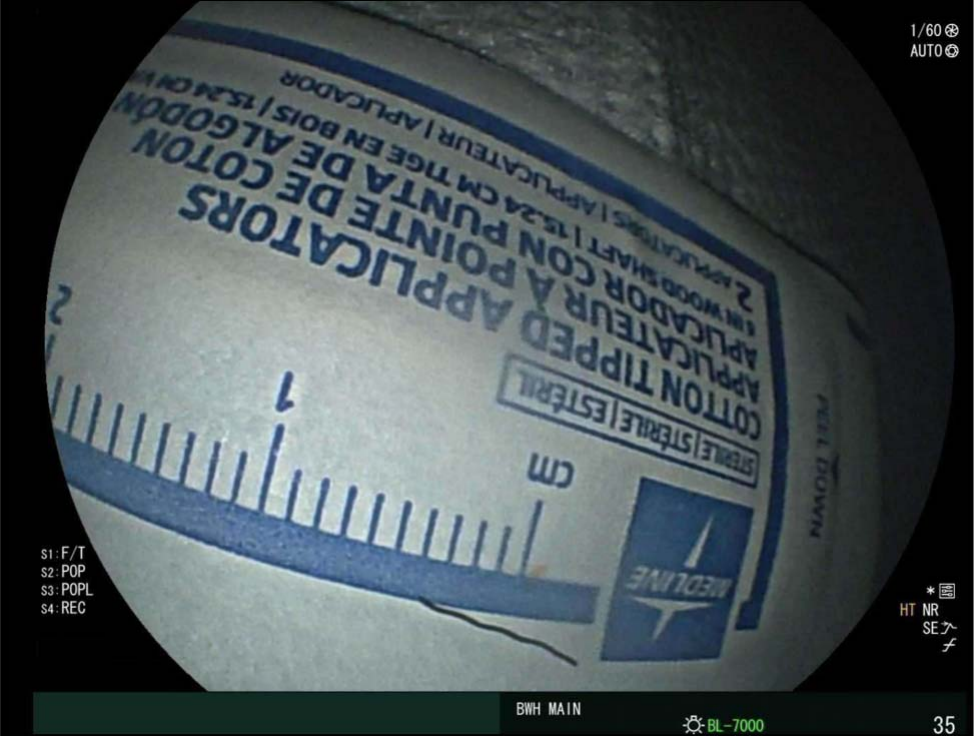
The extracted grill brush bristle (approximately 1 cm).

Pancreatic duct was cannulated and stented, and then biliary cannulation was successful. The lower third of the biliary tree contained a possible filling defect on cholangiogram (Figure 3). A biliary sphincterotomy was performed, and the bile duct was swept 5 times with a biliary extraction balloon (Boston Scientific, Marlborough, MA). This maneuver revealed a metallic-like FB protruding from the major papilla, with its proximal end appearing embedded in the distal bile duct (Figure 4). The FB was carefully extracted using pediatric cold forceps (Boston Scientific, Marlborough, MA) (Figure 5) and was identified as a metallic grill brush bristle. The patient was discharged postprocedure without complications and reported complete resolution of symptoms at her 4-week clinic follow-up.

## DISCUSSION

Our case demonstrated penetration of a sharp FB into both CBD and pancreas—an exceptionally rare occurrence that may present as an unusual cause of chronic abdominal pain. The exact mechanism remains unclear; however, migration of the FB through the papillary opening into the distal CBD and proximal main pancreatic duct is plausible, particularly as the patient had no history of papillotomy or prior surgical manipulation that could have provided direct access to the biliary tree. Previous reports have described FB migration into the CBD via the papilla, especially in cases where soft material impaction at the duodenal papilla facilitated entry.^2,3^

Similarly, Dias et al described ingestion of a metal pin that was found within the CBD and successfully removed by ERCP.^3^ Sinagra et al reported a case in which a screw from a surgical retractor became lodged in the CBD of a patient who had previously undergone left lateral hepatectomy; this FB was also removed endoscopically.^4^ Foreign bodies such as surgical materials and bones are among the most commonly reported causes of impaction in the gastrointestinal tract.^5^ In contrast, grill brush bristles are predominantly described as causing impaction in the oropharynx or leading to esophageal and intestinal perforations.^6–9^

Clinical manifestations can include abdominal pain, fever, bleeding, fistula formation, or secondary bile duct stone development.^10,11^ Perforations typically occur in anatomically fixed regions or areas of luminal narrowing.^12,13^ Reports of gastrointestinal perforation extending into CBD and pancreatic head are limited, thus no standardized guidelines currently exist for managing wire bristle FBs.

In addition, FBs may cause local mucosal damage, leading to edema, necrosis, ulcer formation, and eventually perforation.^2^ The FB localized into the CBD may serve as a nidus for stone formation, which can lead to symptoms of biliary obstruction.^10^ Involvement of the pancreatic duct can trigger acute pancreatitis, whereas transmural perforation may extend into the peritoneal or retroperitoneal cavity, significantly increasing morbidity.

Unintentional ingestion of grill brush bristles has been recognized. Wong et al reviewed 23 cases with varied upper aerodigestive tract presentations.^14^ Dalton et al reported an esophageal perforation requiring surgical intervention, whereas Larsen et al described a small bowel perforation.^15,16^ In 2012, the Centers for Disease Control and Prevention issued a report highlighting injuries related to wire bristle ingestion.^10^

Although computed tomography is commonly used to detect FB, its sensitivity may be limited depending on the object's size and composition. In our case, EUS provided critical diagnostic value. To his end, EUS can also offer additional benefit by precisely localizing the FB in relation to vital structures. If the FB is completely embedded in the pancreatic parenchyma, this would suggest that endoscopic removal could be very difficult or even impossible. Giovanni et al showed how EUS was used to localize a FB within the pancreatic parenchyma, which was subsequently endoscopically removed via a fistulous tract.^2^

In our case, we demonstrated that endoscopic removal is a minimally invasive approach and should be considered as the first-line treatment. Different endoscopic maneuvers can be considered to facilitate safe removal, this can include the use of biliary extraction balloon, pediatric forceps under fluoroscopic guidance, biliary baskets, or cholangioscopy.^17^ However, careful attention is needed to avoid injury to surrounding structures. Furthermore, surgical intervention remains a viable option when endoscopic methods are unsuccessful or not feasible.

This rare case highlights the potential for ingested foreign bodies, such as grill brush bristles, to penetrate both the biliary and pancreatic ducts. Endoscopic ultrasound allows precise localization, whereas ERCP provides an effective, minimally invasive therapeutic option. Together, these modalities underscore the diagnostic and therapeutic value of advanced endoscopic approaches in managing such unusual presentations.

## DISCLOSURES

Author contributions: L.M Assis contributed collecting images and writing, A. Qatomah contributed writing and editing, and D.Ramai contributed organizing the manuscript and language revision. M. Ryou critically reviewed the manuscript for important intellectual content and provided final approval for publication and is the article guarantor.

Financial disclosure: None to report.

Informed consent was obtained for this case report.

## ABBREVIATIONS:

CBD: common bile duct
ERCP: endoscopic retrograde cholangiopancreatography
EUS: endoscopic ultrasound
FB: foreign body
